# Retraction Note: Optical and structural properties of Sn and Ag-doped PbS/PVA nanocomposites synthesized by chemical bath deposition

**DOI:** 10.1038/s41598-024-54118-5

**Published:** 2024-02-14

**Authors:** Ali Fatemi, Tavakkol Tohidi, Kazem Jamshidi-Galeh, Milad Rasouli, Kostya Ostrikov

**Affiliations:** 1 Department of Physics, Azarbaijan Shahid Madani University, Tabriz, Iran; 2 Northwest Research Complex (Bonab), Radiation Applications Research School, Nuclear Science and Technology Research Institute (NSTRI), Tehran, Iran; 3 Department of Physics, Kharazmi University, Tehran, Iran; 4 Department of Physics, Science and Research Branch, Islamic Azad University, Tehran, Iran; 5 School of Chemistry and Physics and QUT Centre for Materials Science, Queensland University of Technology (QUT), Brisbane, Australia

Retraction of: *Scientific Reports* 10.1038/s41598-022-16666-6, published online 28 July 2022

The Editors have retracted this Article.

After the publication of a Correction ^1^, additional concerns were raised about the corrected X-ray diffraction spectra presented in Figure 1.

The Authors repeated the experiments and provided the Editors with the new spectra, but an expert peer review of this data by the journal concluded that the new results were not consistent with those of the published Article and its Correction. The Editors therefore no longer have confidence in this work and its conclusions.

All Authors agree to the retraction and its wording.

## References

[1] Fatemi A, Tohidi T, Jamshidi-Galeh K (2023). Author Correction: Optical and structural properties of Sn and Ag-doped PbS/PVA nanocomposites synthesized by chemical bath deposition. Sci Rep.

